# Opioid analgesics increase incidence of somnolence and dizziness as adverse effects of pregabalin: a retrospective study

**DOI:** 10.1186/s40780-015-0032-5

**Published:** 2015-12-01

**Authors:** Akihiro Ohishi, Yugo Chisaki, Daiki Hira, Kazuki Nagasawa, Tomohiro Terada

**Affiliations:** Department of Pharmacy, Shiga University of Medical Science Hospital, Otsu, Shiga 520-2192 Japan; Department of Environmental Biochemistry, Kyoto Pharmaceutical University, 5 Nakauchi-cho, Misasagi, Yamashina-ku, Kyoto, 607-8414 Japan; Education and Research Center for Clinical Pharmacy, Kyoto Pharmaceutical University, 5 Nakauchi-cho, Misasagi, Yamashina-ku, Kyoto, 607-8414 Japan

**Keywords:** Pregabalin, Adverse effect, Opioid analgesic, Pain relief

## Abstract

**Background:**

Pregabalin, a gabapentinoid, is an adjuvant analgesic for treatment of neuropathic pain, but it has serious adverse effects such as somnolence and dizziness, particularly in elderly patients. Although decreased renal function is considered to the contributing factor for high frequency of these adverse effects in elder patients, only a few systematic clinical investigations, especially for hospitalized patients, have been performed on factors that might affect the incidence of its adverse effects. In this study, we performed a retrospective study on the effect of concomitant drugs on induction of somnolence and dizziness as adverse effects of pregabalin in hospitalized patients.

**Methods:**

The subjects were all pregabalin-administered patients in Shiga University of Medical Science Hospital from September 2010 to September 2012, and the subject number was 195. Multivariate logistic regression analysis was performed to determine predictors of the adverse effects, creatinine clearance, duration of pregabalin therapy, initial and maintenance doses of pregabalin, and concomitant drugs, including hypoglycemic drugs, anti-hypertensive ones, non-steroidal anti-inflammatory ones, opioids and central nervous system depressants, being used as independent variables.

**Results:**

The median initial doses of pregabalin in each renal function group were the same with the case of the defined dose. Although renal function is a well-known factor for prediction of development of adverse effects of pregabalin, we did not detect significant contribution of it. Alternatively, it was demonstrated that concomitant administration of opioids was the significant factor of the incidence of somnolence and dizziness. The first onset date of the adverse effects was frequently detected in the early days of the pregabalin therapy.

**Conclusions:**

The fine tuning of pregabalin dosage schedule based on the renal function appeared to be critical for prevention of development of its adverse effects. Adverse effects tended to develop in the initial phase of pregabalin therapy. Concomitant administration of opioids with pregabalin has the potential to increase the incidence of adverse effects, and thus much more careful attention has to be paid especially in those situations.

## Background

Pain is divided into two phenotypes, nociceptive and neuropathic pain. The former is usually treated with conventional analgesics such as non-steroidal anti-inflammatory drug (NSAID) and opioids according to the WHO’s pain ladder. As for the latter, on the other hand, in addition to conventional analgesics, anti-depressants and anti-convulsants are used as adjuvant ones [1]. However, in the clinical situation, since patients often suffer from both pain phenotypes, they receive combination therapy with conventional and adjuvant analgesics [2–4]. Anti-convulsants, gabapentin and pregabalin are recommended for the first-line therapy in the guidelines for neuropathic pain, and are widely used as adjuvant analgesics for the patients with neuropathic pain, painful diabetic neuropathy, and chemotherapy-induced peripheral neuropathy [5–7]. While the efficacy for pain relief is similar for the two medications, pregabalin has pharmacokinetic/pharmacodynamic advantages compared to gabapentin such as higher affinity for the α2δ subunit of voltage-dependent calcium channels [8, 9], more rapid onset of action, high oral bioavailability and linear pharmacokinetics [10]. After intestinal absorption, pregabalin in the plasma is mainly excreted into the urine as an unchanged drug as in the case of gabapentin, and thus its dose is determined based on renal function indicated by creatinine clearance (CLCr) in patients [10]. Nevertheless, it is well-known that pregabalin causes a lot of adverse effects in the clinical situation. In the interim report of the Japanese post-marketing treatment outcome study on pregabalin (Lyrica^®^), of which most of the patients were outpatients, somnolence and dizziness were found to be high frequency adverse effects in elder patients [11]. In addition, such adverse effects are reported to cause tumbling and traffic accidents [12]. Thus, the Pharmaceuticals and Medical Devices Agency of Japan released a notice regarding use of pregabalin in elder patients in July 2012. On the other hand, it is well-known that drug-drug interaction is one of critical factors for induction of adverse effects of either or both drugs, but there are only a few systematic clinical studies on the factors which affect development of adverse effects of pregabalin [13, 14].

In this study, therefore, we performed a retrospective study to determine whether drug-drug interaction of pregabalin with concomitantly administered drugs was one of the factors for induction of the adverse effects of pregabalin in hospitalized patients.

## Methods

### Participants

This study was approved by the ethics committee of Shiga University of Medical Science Hospital (#24-125). The subjects were all pregabalin-administered patients in Shiga University of Medical Science Hospital from September 2010 to September 2012 except for exclusion criteria (described below), and the subject number was 195. In these periods, pregabalin was prescribed from the several clinical departments not only for the treatment of cancer pain but also for that of neuropathic pain, painful diabetic neuropathy, post-herpetic neuralgia, fibromyalgia, post-operative neuropathy and chemotherapy-induced peripheral neuropathy. The departments were as followed: orthopedic surgery, hematology, dermatology, respiratory medicine, thoracic surgery, urology, neurology, otorhinolaryngology-head and neck surgery, endocrinology and metabolism, neurosurgery, gastrointestinal surgery, female pelvic surgery and reproductive medicine, maternal and fetal medicine, pain management clinic, nephrology and diabetes, cardiovascular surgery, general surgery, physical medicine and rehabilitation, gastroenterology, cardiology, emergency and I.C.U. and psychiatry. The occurrences of somnolence or dizziness during the administration of pregabalin were collected from medical charts, which were recorded by medical staffs based on interviews with the patients in daily clinical practice. Although, due to the data collection method, the severity of the adverse effects could not be evaluated, they might be regarded as grade ≥1 of the Common Terminology Criteria for Adverse Events version 4.0 because the data were based on patients’ words. The patients who used pregabalin on an as-needed basis, who were under 18 years old, who were not prescribed pregabalin for the first time and who did not have complete data for analysis were excluded.

### Collection of patient data

The data collected included basic demographic information such as age, gender, serum creatinine level, duration of pregabalin therapy, initial and maintenance doses of pregabalin, administration protocol for pregabalin that followed the defined one in the package insert, and concomitant drugs, which included NSAID, hypoglycemic drugs, anti-hypertensive drugs, opioids and central nervous system (CNS)-depressants excluding opioids, which might contribute to the increase in the incidence of somnolence and dizziness as adverse effects of pregabalin. Duration of the pregabalin therapy of the patient who prescribed pregabalin over the data collection period was regarded as terminated in the last day of the data collection period. The CLCr in patients were calculated using the Cockcroft-Gault equation.

### Statistical analysis

Multivariate logistic regression analysis was performed to determine predictors of adverse effects, the following independent variables being included; CLCr, duration of pregabalin therapy, initial dose, maintenance dose, and defined/undefined doses of pregabalin, and concomitant drugs. The CLCr values were significantly correlated with age, and thus age was not used as an independent variable to avoid multicollinearity. Statistical analysis was performed using StatView 5.0 software (SAS Institute Inc.), a *p*-value of 0.05 or less being considered statistically significant.

## Results

**Fig. 1:**
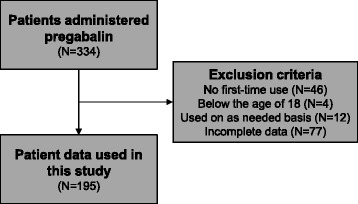
Schematic illustration of the exclusion criteria in this study

The basic characteristics of the 195 patients analyzed in this study are summarized in Table 1. A total 334 patients were recruited, and after exclusion of 139 patients based on the criteria described under Methods, complete data were obtained for 195 patients (Fig. 1; 114 males and 81 females), whose median age, initial and maintenance doses were 67.0 years old, 75 mg/day and 150 mg/day, respectively. When the patients were classified as to their renal function, 127 patients (65.1 %) with CLCr values of more than 60 mL/min exhibited normal renal function, 55 (28.2 %) with more than 30 to less than or equal 60 mL/min mild renal dysfunction, and 13 (6.7 %) with less than or equal 30 mL/min severe renal dysfunction. As for adverse effects, there was no apparent difference in the incidence of somnolence and dizziness among normal renal function, and mild and severe renal dysfunction. The median initial doses of pregabalin in patients with normal renal function, and mild and severe renal dysfunction being 150, 75 and 50 mg/day, respectively (Table 2). These median doses are the same with the defined initial doses for each patient based on the renal function.

**Table 1:** Characteristics of study population

Demographic factors	N	Median (range)
Male	114 (59.5 %)	
Female	81 (41.5 %)	
Age		67.0 (20–90)
Dose of pregabalin		Median (range)
Initial dose, mg/day		75 (25–450)
Maintenance dose, mg/day		150 (25–450)
Adverse effects	N	
Patients who developed one or both of side effects	63 (32.3 %)	
Patients who developed somnolence	47 (24.1 %)	
Patients who developed dizziness	34 (17.4 %)

**Table 2:** Renal function and prescribed doses

CLCr (mL/min)	N	Median of initial dose (mg/day, range)	Defined initial dose (mg/day)	Developed adverse effects	Developed adverse effects with over defined initial dose
>60	127	150 (25–450)	150	41 (32.3 %)	1 (2.4 %)
>30–≤60	55	75 (25–150)	75	17 (30.9 %)	8 (47.1 %)
≤30	13	50 (25–150)	25 or 50	5 (38.5 %)	2 (40.0 %)

Distribution of the number of patients whose duration of pregabalin therapy for 2 week or less, more than 2 weeks to 1 month or less, more than 1 to 2 months or less and more than 2 months were 21 (10.8 %), 30 (15.4 %), 36 (18.5 %) and 108 (55.4 %), respectively (Table 3). There was no apparent difference in ratios of the patients who developed adverse effects among each period. However, first onset date of the adverse effects from the start of pregabalin medication was the most frequent within 2 weeks (Table 4).

**Table 3:** Characteristics of patients who developed dizziness or somnolence

Duration of pregabalin therapy	N	Developed somnolence or dizziness
≤2 weeks	21 (10.8 %)	8 (38.1 %)
>2 weeks–≤1 month	30 (15.4 %)	7 (23.3 %)
>1 month–≤2 months	36 (18.5 %)	11 (30.6 %)
>2 months	108 (55.4 %)	37 (34.3 %)
Median (day, range)	78 (1–740)	
Dosage	N	Developed somnolence or dizziness
Within the defined initial dosage range	166 (85.1 %)	52 (31.3 %)
Over the defined initial dosage range	29 (14.9 %)	11 (37.9 %)
Concomitant drugs with pregabalin	N	Developed somnolence or dizziness
Oral hypoglycemic drugs	28 (14.4 %)	6 (21.4 %)
Antihypertensive drugs	55 (28.2 %)	15 (27.2 %)
NSAID	64 (32.8 %)	15 (23.4 %)
Opioid pain relievers	54 (27.7 %)	25 (46.3 %)
CNS- depressants	31 (15.9 %)	9 (29.0 %)

**Table 4:** Distribution of the first onset date of adverse effects from the start of pregabalin medication

First onset date of adverse effects	N (% of adverse effect developed patients)
≤2 weeks	46 (73.0 %)
>2 weeks–≤1 month	9 (14.3 %)
>1 month–≤2 months	1 (1.6 %)
>2 months	7 (11.1 %)

As for the initial doses of pregabalin (Table 3), 166 patients (85.1 %) were prescribed it in the defined dose range, while 29 (14.9 %) received more than the defined doses, i.e., about 2-fold greater. The incidence of adverse effect tended to be higher in patients who were prescribed over the defined dose than in patients whose doses were within the defined one.

As shown in Table 3, 115 patients received concomitant drugs with pregabalin, hypoglycemic drugs, anti-hypertensive drugs, NSAIDs, opioids and CNS-depressants being prescribed to 28 (14.4 %), 55 (28.2 %), 64 (32.8 %), 54 (27.7 %) and 31 (15.9 %) patients, respectively, and the numbers of patients with somnolence and dizziness being 6 (21.4 %), 15 (27.2 %), 15 (23.4 %), 25 (46.3 %) and 9 (29.0 %), respectively.

In Table 5, the odds ratios derived from multivariate logistic regression model are summarized. Among ten factors evaluated, concomitant use of opioids with pregabalin exhibited the statistically significant higher rate of induction of somnolence and dizziness compared to without them (odds ratio (OR) = 2.700, 95 % confidence interval (CI) = 1.282–5.689, *p* = 0.009). Thus, among these drugs, the concomitant administration of opioids with pregabalin might have the high potential to induce somnolence and dizziness in patients. Incidence of somnolence and dizziness in patients who were prescribed pregabalin without opioids were 19.9 and 14.9 %, and who used pregabalin and opioids concomitantly were 35.2 and 24.1 %, respectively (Table 6). Four kinds of opioids were used with pregabalin in 54 patients. Among them, oxycodone showed the highest incidence of adverse effects (47.8 %). Other two opioids also induced the adverse effects in more than 25 % patients, but their sample size is too small to analyze.

**Table 5:** Predictive value for each factor

Factor	OR^a^	95 % CI^b^	*P* value
CLCr	0.990	0.979–1.001	0.080
Duration of pregabalin therapy	0.999	0.998–1.001	0.521
Initial dose	1.000	0.992–1.008	0.945
Maintenance dose	1.003	0.998–1.009	0.187
Over the defined initial dose range	0.739	0.246–2.220	0.590
Hypoglycemic drug	0.607	0.221–1.670	0.333
Antihypertensive drug	0.707	0.340–1.471	0.354
NSAID	0.758	0.370–1.554	0.450
Opioid analgesic	2.700	1.282–5.689	0.009
CNS- depressant	0.622	0.248–1.562	0.312

^a^ *OR* odds ratio

^b^ *CI* confidence interval

**Table 6:** Prescribed opioids and the incidence of somnolence and dizziness

Concomitant use of opioid with pregabalin	N	Developed somnolence	Developed dizziness
Non-prescribed patients	141	28 (19.9 %)	21 (14.9 %)
Prescribed patients	54	19 (35.2 %)	13 (24.1 %)
Concomitant opioids	N	Developed somnolence or dizziness	
Fentanyl	26	7 (26.9 %)	
Oxycodone	23	11 (47.8 %)	
Tramadol	18	7 (38.9 %)	
Morphine	4	0 (0 %)	

## Discussion

A high incidence of somnolence and dizziness was detected in the patients who had co-administration of pregabalin and opioids. There was a tendency that adverse effects were frequently developed in the early days of pregabalin medication.

In the previous studies, the elderly patients tended to have high serum concentrations of pregabalin [15] and a high incidence of adverse effects of pregabalin [11], which were reasonable considering their decreased renal function. However, in the present study, there was no difference in the incidence of adverse effects among each renal function (Table 2). Although there seems to be a discrepancy between the previous study and ours, it is considered to be explained by the fine tuning of the doses based on the renal function of the hospitalized patients as described below. The average of age and ratios of the patients’ renal function in the interim report of the drug use investigation of pregabalin are almost the same with the population of this study [11]. The major difference between two studies is patient population, that is to say, population of our study is inpatients, in contrast to outpatients in the previous report. Hospitalized patients were received their physical management more strictly compared to the case of clinical trials. During hospitalization, patients were assessed their physical condition such as renal function and efficacy/adverse effects of pregabalin in detail every day. The same as the case of our study, the retrospective study on pregabalin in hospital inpatients demonstrated that the renal function of the patients was not a significant risk factor for induction of somnolence and dizziness [13, 14].

Kanbayashi et al. found that prolonged duration of pregabalin therapy significantly increased the incidence of somnolence [14]. On the other hand, in the present study, duration of the pregabalin therapy was not detected as the significant factor, and onsets of the adverse effects were frequently detected in the early days of pregabalin medication. Although these results are opposite to Kanbayashi’s one, it is reasonable that the adverse effects developed frequently in the initiation phase of pregabalin therapy, because this phase is in the duration of which titrate an appropriate dosage of pregabalin for each patient. Additionally, maintenance dose of the pregabalin was not identified as the significant factor for incidence of adverse effects, either as with the report of Kanbayashi’s one. From these results, it is conceivable that the dosage of pregabalin was titrated with the monitoring of the condition of adverse effects, according to the defined usage of the pregabalin, which suggested that start medication from low doses and escalate the dose with the monitoring of the adverse effect development. The present study reported the frequent development of adverse effects of pregabalin in the initial phase of the therapy for the first time. This finding supports the defined usage of the pregabalin which suggested that the dose should be escalated from low dosage. Together, fine tuning of pregabalin dosing schedules for each patient based on their physical conditions is critical for its safe and effective use.

Very recently, Watanabe et al. reported that concomitant use of opioid was the risk factor of the somnolence and dizziness in hospitalized patients [13]. This correlates significantly with current result, although the number of assessed patients were not so large (*n* = 65), as compared to the present study (*n* = 195). These findings indicate that concomitant use of pregabalin and opioids is a common risk factor for somnolence and dizziness, and this finding is clinically important information. This finding will be robust by the large-scale investigation and meta-analysis.

Opioids have the same adverse effects, somnolence and dizziness, as pregabalin, and thus we can not define the underlying mechanism. In meta-analysis, median occurrence rates of somnolence and dizziness in opioid-treated patients were 21 % (range: 10–39 %) and 22 % (range: 10–37 %), in pregabalin-treated patients were 13.7 % (range: 5.7–25.7 %) and 24.1 % (range: 8.7–35.5 %), respectively [16, 17]. These data suggest that incidences of somnolence and dizziness of both drugs are the same level. In the present study, incidences of somnolence and dizziness of patients who used pregabalin without opioids were 19.9 and 14.9 %, respectively. Since we have not collected the data of patients who use opioids without pregabalin, we can not evaluate the incidence of somnolence and dizziness of them in those patients. Both incidences of somnolence and dizziness of patients who used pregabalin without opioids were in the ranges of the result of meta-analysis. However, in the present study, incidences of the adverse effects in patients who used pregabalin and opioids concomitantly (somnolence: 35.2 %, dizziness: 24.1 %) were higher than those of patients who used pregabalin without opioids. This result suggested that increased incidence of adverse effects might be caused by additive effect of opioids.

The target of pregabalin is the α_2_-δ1 auxiliary subunits of voltage-dependent calcium channels, while that of opioids is μ-opioid receptors, both of which activate the descending noradrenergic system [18, 19]. A target of gabapentin is reported to be the α_2_-δ1 subunits as pregabalin [20, 21]. It appears that co-administration of gabapentin and opioids leads to great anti-neuropathic pain efficacy without an apparent increase of adverse effects [3, 4, 22]. This preferable interaction between gabapentin and opioids is considered to be due to activation of the descending noradrenergic system [23], but whether their interaction is additive or synergetic remains unknown, and there has been a report that concomitant administration of morphine with gabapentin results in their pharmacokinetic interaction, leading to an increased serum concentration of gabapentin [24]. Since pregabalin appears not to be metabolized and to have no effect on functional expression of cytochrome P450 isozymes [25], it is considered that there is no or only a negligible possibility of a pharmacokinetic interaction between pregabalin and opioids. On the other hand, it has been reported that pregabalin exhibits 6-fold greater affinity for presynaptic calcium channels than gabapentin [8, 9], and thus co-administration of pregabalin and opioids might induce their adverse effects to greater levels than in the case of gabapentin. However, as described above, the severity of the adverse effects have not evaluated and this study was a retrospective one, and thus, precise rates of the incidence of adverse effects were unclear. To clarify the mechanism underlying this interaction, detail investigations are needed.

Dou et al. (2014) investigated the efficacy and safety of pregabalin in patients undergoing morphine therapy by double-blind, randomized, placebo-controlled crossover study [26]. They showed that combination use of the pregabalin and morphine increased the incidence of somnolence and dizziness, as like in the present study. On the other hand, they also showed that concomitant use of pregabalin contributes to reduce the dose of morphine with the same pain-relief efficacy, compared to that in morphine-monotherapy. These results suggest that although combination use of the two drugs increases the incidence of the adverse effects, their concomitant use could lead to avoid the dose-limiting adverse effects such as nausea, constipation and vomiting caused by morphine. Thus, concomitant use of pregabalin and opioids should be performed with the careful consideration of the risks and benefits.

All these independent investigations clearly demonstrated that concomitant administration of opioids with pregabalin has the potential to increase the incidence of adverse effects, and that fine tuning of pregabalin dosing schedules for each patient based on their physical conditions is critical for its safe and effective use. Further analysis based on larger-scale database may provide more exact information for risk factors.

In cancer chemotherapy including peripheral neuropathy-inducible agents such as oxaliplatin [27], pregabalin is frequently prescribed to the patients in addition to opioids for prevention of somatic/visceral pain [28, 29]. Considering increase of numbers of the outpatients receiving cancer chemotherapy in hospital, there is a possibility that pregabalin is unexpectedly prescribed to opioid-administered patients in clinics. In such outpatients, there is an increasing possibility of development of somnolence and dizziness as the adverse effects of pregabalin/opioid, resulting in falling and tumbling of them, because pharmaceutical care is less than the case of inpatients. To avoid such unexpected situation, information sharing among medical staffs is crucial, and one of the effective ways for its prevention is considered to use of medication notebook [30, 31].

## Conclusion

In conclusion, the present study revealed that concomitant administration of opioids with pregabalin increased the incidence of somnolence and dizziness in the patients, and strict dosage schedule management based on the renal function of individual patients is critical for safe and effective neuropathic pain treatment using pregabalin. Our findings are supported by recent other independent studies, suggesting that severe potential risks may be included for the co-administration of opioids with pregabalin. For safer pharmacotherapy of pregabalin, concomitant administration of opioids, in addition to age and renal function, should be paid attention as risk factors.

## Abbreviations

CI: confidence interval
CLCr: creatinine clearance
CNS: central nervous system
NSAID: non-steroidal anti-inflammatory drug
OR: odds ratio

## References

[1] Kong VKF, Irwin MG (2009). Adjuvant analgesics in neuropathic pain. Eur J Anaesthesiol.

[2] Caraceni A, Zecca E, Bonezzi C, Arcuri E, Yaya Tur R, Maltoni M, et al. Gabapentin for neuropathic cancer pain: a randomized controlled trial from the Gabapentin Cancer Pain Study Group. J Clin Oncol. 2004;22:2909–17. 10.1200/JCO.2004.08.141 15254060

[3] Keskinbora K, Pekel AF, Aydinli I (2007). Gabapentin and an opioid combination versus opioid alone for the management of neuropathic cancer pain: A randomized open trial. J Pain Symptom Manage.

[4] Caraceni A, Zecca E, Martini C, De Conno F (1999). Gabapentin as an adjuvant to opioid analgesia for neuropathic cancer pain. J Pain Symptom Manage.

[5] Dworkin RH, O’Connor AB, Backonja M, Farrar JT, Finnerup NB, Jensen TS, et al. Pharmacologic management of neuropathic pain: evidence-based recommendations. Pain. 2007;132:237–51. 10.1016/j.pain.2007.08.033 17920770

[6] Attal N, Cruccu G, Baron R, Haanpää M, Hansson P, Jensen TS, et al. EFNS guidelines on the pharmacological treatment of neuropathic pain: 2010 revision. Eur J Neurol. 2010;17:1113–23. 10.1111/j.1468-1331.2010.02999.x 20402746

[7] Tan T, Barry P, Reken S, Baker M (2010). Pharmacological management of neuropathic pain in non-specialist settings: summary of NICE guidelines. Brit Med J.

[8] Marais E, Klugbauer N, Hofmann F. Calcium channel α_2_δ subunits-structure and gabapentin binding. Mol Pharmacol. 2001;59:1243–8. 10.1124/mol.59.5.1243 11306709

[9] Li Z, Taylor CP, Weber M, Piechan J, Prior F, Bian F, et al. Pregabalin is a potent and selective ligand for α_2_δ-1 and α_2_δ-2 calcium channel subunits. Eur J Pharmacol. 2011;667:80–90. 10.1016/j.ejphar.2011.05.054 21651903

[10] Randinitis EJ, Posvar EL, Alvey CW, Sedman AJ, Cook JA, Bockbrader HN (2003). Pharmacokinetics of pregabalin in subjects with various degrees of renal function. J Clin Pharmacol.

[11] Ogawa S, Komatsu M, Ohno S, Yamane H, Hayakawa K (2013). Interim report of drug use investigation of pregabalin (Lyrica®). Prog Med.

[12] Otaka Y (2015). Fall prevention in older people: present and future perspectives. Jpn J Fall Prevention.

[13] Watanabe M, Mita K, Nakamura H, Tanaka T, Mihara K, Ono H (2014). Risk factors associated with dizziness and somnolence in hospitalized patients receiving pregabalin. Jpn J Pharm Health Care Sci.

[14] Kanbayashi Y, Onishi K, Hosokawa T (2014). Factors predicting adverse events associated with pregabalin administered for neuropathic pain relief. Pain Res Manag.

[15] May TW, Rambeck B, Neb R, Jürgens U (2007). Serum concentrations of pregabalin in patients with epilepsy: the influence of dose, age, and comedication. Ther Drug Monit.

[16] Papaleontiou M, Henderson Jr CR, Turner BJ, Moore AA, Olkhovskaya Y, Amanfo L, et al. Outcomes associated with opioid use in the treatment of chronic noncancer pain in older adults: a systematic review and meta-analysis. J Am Geriatr Soc. 2010;58:1353–69. 10.1111/j.1532-5415.2010.02920.x PMC3114446 20533971

[17] Zhang SS, Wu Z, Zhang LC, Chen RP, Huang YH, Chen H (2015). Efficacy and safety of pregabalin for treating painful diabetic peripheral neuropathy: a meta-analysis. Acta Anaesthesiol Scand.

[18] Hayashida K, Obata H, Nakajima K, Eisenach JC (2008). Gabapentin acts within the locus coeruleus to alleviate neuropathic pain. Anesthesiology.

[19] Jones SL, Gebhart GF (1988). Inhibition of spinal nociceptive transmission from the midbrain, pons and medulla in the rat: activation of descending inhibition by morphine, glutamate and electrical stimulation. Brain Res.

[20] Field MJ, Cox PJ, Stott E, Melrose H, Offord J, Su T, et al. Identification of the α_2_-δ-1 subunit of voltage-dependent calcium channels as a molecular target for pain mediating the analgesic actions of pregabalin. Proc Natl Acad Sci U S A. 2006;103:17537–42. 10.1073/pnas.0409066103 PMC1859964 17088553

[21] Li CY, Zhang XL, Matthews EA, Li KW, Kurwa A, Boroujerdi A, et al. Calcium channel α_2_δ_1_ subunit mediates spinal hyperexcitability in pain modulation. Pain. 2006;125:20–34. 10.1016/j.pain.2006.04.022 PMC1635965 16764990

[22] Gilron I, Bailey JM, Tu D, Holden RR, Weaver DF, Houlden RL (2005). Morphine, gabapentin, or their combination for neuropathic pain. N Engl J Med.

[23] Tanabe M, Takasu K, Takeuchi Y, Ono H (2008). Pain relief by gabapentin and pregabalin via supraspinal mechanisms after peripheral nerve injury. J Neurosci Res.

[24] Eckhardt K, Ammon S, Hofmann U, Riebe A, Gugeler N, Mikus G (2000). Gabapentin enhances the analgesic effect of morphine in healthy volunteers. Anesth Analg.

[25] Ben-Menachem E (2004). Pregabalin pharmacology and its relevance to clinical practice. Epilepsia.

[26] Dou Z, Jiang Z, Zhong J. Efficacy and safety of pregabalin in patients with neuropathic cancer pain undergoing morphine therapy. Asia Pac J Clin Oncol. 2014, *in Press.* 10.1111/ajco.12311 25530068

[27] Sakurai M, Egashira N, Kawashiri T, Yano T, Ikesue H, Oishi R (2009). Oxaliplatin- induced neuropathy in the rat: involvement of oxalate in cold hyperalgesia but not mechanical allodynia. Pain.

[28] Nishihara M, Arai YC, Yamamoto Y, Nishida K, Arakawa M, Ushida T, et al. Combinations of low-dose antidepressants and low-dose pregabalin as useful adjuvants to opioids for intractable, painful bone metastases. Pain Physician. 2013;16:E547–552. 24077205

[29] Vorobeychik Y, Gordin V, Mao J, Chen L (2011). Combination therapy for neuropathic pain: a review of current evidence. CNS Drugs.

[30] Ojima F, Sakurai K, Itoh J, Okazaki C, Takeda N, Handa M, et al. Effectiveness of medication notebooks distributed to health insurance pharmacies in Yamagata prefecture. Jpn J Pharm Health Care Sci. 2005;31:290–4.

[31] Ojima F, Handa M, Takeda N, Sakurai K, Itoh J, Okazaki C, et al. Analyzing the usefulness of medication notebooks at hospital pharmacies in Yamagata prefecture. J Jpn Soc Hosp Pharm. 2005;41:845–7.

